# Evaluation of a Web-Based Self-Management Program for Patients With Cardiovascular Disease: Explorative Randomized Controlled Trial

**DOI:** 10.2196/17422

**Published:** 2020-07-24

**Authors:** Marscha M Engelen, Sandra van Dulmen, Saskia Puijk-Hekman, Hester Vermeulen, Maria WG Nijhuis-van der Sanden, Sebastian JH Bredie, Betsie GI van Gaal

**Affiliations:** 1 IQ Healthcare Radboud Institute for Health Sciences Radboud university medical center Nijmegen Netherlands; 2 Department of Primary and Community Care Radboud Institute for Health Sciences Radboud university medical center Nijmegen Netherlands; 3 Netherlands Institute for Health Services Research Utrecht Netherlands; 4 Faculty of Health and Social Sciences University of South-Eastern Norway Drammen Norway; 5 Institute of Nursing Faculty of Health and Social Studies HAN University of Applied Sciences Nijmegen Netherlands; 6 Department of Internal Medicine Radboud university medical center Nijmegen Netherlands

**Keywords:** explorative randomized controlled trial, cardiovascular diseases, self-management, eHealth support programs, internet, lifestyle, nursing

## Abstract

**Background:**

Web-based self-management programs have the potential to support patients with cardiovascular disease (CVD) in their self-management (eg, by focusing on behavior change and improving physical activity). The intervention mapping framework was used to develop a web-based program called Vascular View. The Vascular View program contained 6 modules (coping with CVD, setting boundaries, lifestyle, healthy nutrition, being physically active, interaction with health professionals) aiming to increase self-management behavior by tailoring to the perceived problems and (support) needs of patients after CVD.

**Objective:**

The aim was to test the effectiveness of Vascular View before embarking on a full-scale randomized clinical trial (RCT) by evaluating the potential effectiveness and effect sizes of the Vascular View program and identifying outcome measures most likely to capture the potential benefits.

**Methods:**

An explorative RCT was performed. Both control and intervention groups received care as usual and, in addition, the intervention group received 12 months of access to a web-based self-management program. Assessment occurred at baseline, 6 months, and 12 months. Outcome measures included general patient-reported outcome measurements: Illness Perception Questionnaire (IPQ), Rand-36, Patient Activation Measure, and patient self-efficacy. Module-specific patient-reported outcome measurements were Beliefs about Medicines Questionnaire, International Physical Activity Questionnaire, Dutch Healthy Diet Index, Fagerström Test for Nicotine Dependence (FTND), Alcohol Use Disorders Identification Test, and Perceived Efficacy in Patient-Physician Interaction. Linear mixed models for repeated measures using intention-to-treat and per-protocol analysis were applied to study differences between the patients in the intervention and control groups. Floor and ceiling effects were explored to give insight into the outcome measures most likely to capture the potential benefits.

**Results:**

A total of 105 patients in the control group and 103 patients in the intervention group participated in the study. A positive direction of change between baseline and 12 months was shown for most outcome measurements in favor of the intervention group, of which 2 out of 10 outcomes showed a significant effect: attribution of cause of the disease to risk factors and immunity factors (IPQ) and dependency of nicotine (FTND). Floor and ceiling effects were seen in the IPQ, Rand-36, and the self-efficacy questionnaire.

**Conclusions:**

No conclusion for the efficacy of the Vascular View program or selection of outcome measurements can be taken yet. A process evaluation will be conducted to gain thorough insight into the working elements of the program, patient needs in eHealth, and the use of the program by patients. This can determine for whom web-based self-management programs will work and help to adapt the program.

**Trial Registration:**

Dutch Trial Register NTR5412; https://www.trialregister.nl/trial/5303

**International Registered Report Identifier (IRRID):**

RR2-10.2196/resprot.6352

## Introduction

Cardiovascular diseases (CVDs) are the leading cause of death worldwide, and survivors of CVD are at high risk for a secondary CVD event [1]. Prevention of secondary CVD events can be influenced by focusing on the interaction of behavioral risk factors (lifestyle components), metabolic risk factors (hypertension, high blood glucose levels, raised blood lipids, and overweight), and other risk factors (eg, advancing age, sex, stress, and depression) [1]. Managing these factors poses high demands on patient self-management skills. Despite support from health care professionals, patients have trouble managing their CVD and its consequences in daily life themselves [2]; they experience disease-related problems such as dealing with the symptoms, treatment, physical and psychological consequences, and recommended lifestyle changes.

Self-management of chronic illness has been widely recognized as a way to support patients in achieving the best possible quality of life with their chronic condition [3,4]. Secondary prevention programs targeting self-management through risk factors and (lifestyle) behavior change have been associated with reduced mortality, reduction of repeated cardiac events, and improved health-related quality of life [5,6]. However, attendance rate in cardiac rehabilitation programs remains less than 50% worldwide [7,8] due to barriers like lack of transportation, embarrassment of participation, a dislike of group environments, and work or domestic commitments [9,10]. The use of web-based self-management programs could be a remedy to these barriers, since these programs have the potential to reach a large group of patients and diminish barriers because of the possibility of accessing programs anytime and anywhere and providing anonymity. Buys and colleagues [11] found that patients with CVD showed a high interest in support through the internet (77%) and mobile phones (68%).

Recent systematic reviews have shown the high potential of electronic health (eHealth) interventions for CVD prevention [12-15]. Widmer and colleagues [14] demonstrated significant reduction on CVD outcomes due to digital health interventions (relative risk –0.61, 95% CI 0.46-0.80; *P*<.001; *I*^2^=22%). Another systematic review studied behavioral change by using mobile health interventions: studies aiming to improve physical activity (n=2) or medication adherence (n=3) showed positive effects, but no effects were seen in studies aiming to decrease smoking (n=1) or change diets (n=1) [12]. Duff and colleagues [15] investigated the use of behavior change techniques (BCTs) in eHealth interventions for improving physical activity for patients with CVD: 8 of the 15 interventions showed significant improvements, while in 5 of the 15 studies the intervention group scored equal to the control group.

Not all studies recognize the potential of eHealth interventions, and demonstrate more questionable results. Hamine and colleagues [16] evaluated the effectiveness of mobile health (mHealth) interventions in supporting adherence of patients to chronic disease management (including CVD). Out of 41 randomized controlled trials (RCTs) that measured the effects of mHealth interventions on disease-specific clinical outcomes, significant improvements between groups were reported in only 16 studies (39%). Specific for cardiovascular risk factors, another review showed that the use of multiple modifiable internet lifestyle interventions in primary or secondary care is not superior to usual care [17]. Although 4 out of 9 studies demonstrated significant improvement in various risk factors, clinical relevance of these differences is questionable [17]. Inconsistent evidence is often due to limitations in the intervention and research design and a lack of power in the studies [18]. Limitations like small sample sizes, unclear description of intervention characteristics, short duration, and selection bias were noted in review studies [12,13].

To reduce the risk of limitations, we used the intervention mapping (IM) framework to develop a comprehensive, multicomponent web-based self-management program for patients with CVD called Vascular View (Vaat in Zicht in Dutch). IM contains 6 steps to design, implement, and evaluate an intervention based on the foundation of theoretical, empirical, and practical information [19]. The program Vascular View aims to increase self-management behavior tailored to the perceived problems and (support) needs of patients after CVD diagnosis [20]. Each of the 6 modules has a specific goal based on determinants of the I-change model [21]. For each of these selected determinants, BCTs were selected [22]. A detailed description of the development and content of Vascular View has been described elsewhere [20].

The last step of IM (step 6) is evaluation. This paper describes the testing of the effectiveness of Vascular View before embarking on a full-scale RCT. This explorative RCT study in patients with CVD aims to evaluate the potential effectiveness and effect sizes of the Vascular View program on 10 patient outcome measures and identify the outcome measures most likely to capture the potential benefits of the Vascular View program.

## Methods

### Design

An explorative RCT was conducted at four outpatient clinics (cardiology, internal medicine, neurology, and vascular surgery) at Radboud University Medical Center in the Netherlands. To explore the efficacy of the web-based self-management program and identify suitable outcome measures, questionnaire data of patients allocated to the intervention and control groups were compared at 6 and 12 months after baseline on 10 outcome measurements related to the performance objectives of the intervention. Table 1 shows the modules, performance objectives, and related outcomes of the intervention. Since all outcomes are targeted through the intervention, positive effects are expected on all outcomes. The trial is registered in the Dutch Trial Register [NTR5412]. The medical ethics committee of Arnhem-Nijmegen in the Netherlands approved this study (registration number: 2015/1908).

**Table 1:** Modules, performance objectives, determinants, and related outcomes of the intervention.

Module	Performance objectives	Determinants	Module-specific outcomes
Coping with CVD^a^ (3 sessions)	Patients have insight into CVD and accompanying symptoms and consequences. Patients cope with CVD and accompanying symptoms and consequences. Patients cope with (changed) sexuality and intimacy. Patients cope with stress in daily life. Patients cope with fear and emotions related to CVD. Patients cope with pain related to CVD. Patients adhere to medication instructions.	Knowledge, awareness, risk perception, attitude, self-efficacy, subjective norm, intention, action plans	BMQ^b^, Self-efficacy (subscale: acceptation)
Setting boundaries in daily life (4 sessions)	Patients set boundaries. Patients adapt to changed circumstances. Patients ask for support from partner, relatives, and social environment. Patients cope with changed roles in family, job, and/or society. Patients are able to resume activities within their own possibilities.	Knowledge; awareness, attitude, self-efficacy, subjective norm	Self-efficacy (subscale: social environment), Self-efficacy (subscale: setting boundaries)
Lifestyle (4 sessions)	Patients refrain from tobacco use. Patients refrain from (harmful) alcohol use.	Knowledge, awareness, attitude, self-efficacy, subjective norm, intention, habits, skills	FTND^c^, AUDIT^d^, Self-efficacy (subscale: smoking), Self-efficacy (subscale: alcohol)
Healthy nutrition (3 sessions)	Patients eat healthy.	Knowledge, awareness, attitude, self-efficacy, subjective norm, intention, habits, skills	DHD^e^, Self-efficacy (subscale: diet)
Being physically active in a healthy way (3 sessions)	Patients are physically active.	Knowledge, awareness, attitude, self-efficacy, subjective norm, intention, habits, skills	IPAQ^f^, Self-efficacy (subscale: physical activity)
Interaction with health professionals (4 sessions)	Patients interact with health professionals.	Knowledge, awareness, attitude, self-efficacy, subjective norm	PEPPI-5^g^, Self-efficacy (subscale interaction)

^a^CVD: cardiovascular disease.

^b^BMQ: Beliefs about Medicine Questionnaire.

^c^FTND: Fägerstorm Test for Nicotine Dependence.

^d^AUDIT: Alcohol Use Disorders Identification Test.

^e^DHD: Dutch Health Diet Index.

^f^IPAQ: International Physical Activity Questionnaire.

^g^PEPPI-5: Perceived Efficacy in Patient-Physician Interactions.

### Participants

In the third quarter of 2015, 600 consecutive patients who had visited the outpatient clinic because of an established cardiovascular event were invited by the treating medical specialist to participate in this study. Inclusion criteria were (1) a cardiovascular disease (myocardial infarction, cerebrovascular disease [stroke included], peripheral artery disease, or combination); (2) a CVD event within 2 months to 1 year before start of the study; (3) aged 18 years or older; (4) able to read and understand Dutch; and (5) have access to a computer, internet, and an email account. Patients with a psychiatric disorder were excluded. Patients received information about the content and aim of the study, a short questionnaire to assess the inclusion criteria, and an informed consent form from the medical specialist via postal letter. Patients were asked to sign and return the informed consent form and completed questionnaire to the researcher (ME). When patients agreed to participate and were eligible, they received an invitation to complete the online questionnaire baseline data collection.

### Randomization

Randomization took place after the baseline measurement and was stratified for four patient diagnoses: myocardial infarction, cerebrovascular disease (stroke included), peripheral artery disease, and aneurysm. The most recent diagnosis was used in randomization for patients with comorbidity in CVD. A blinded and independent statistician executed the randomization using SAS version 9.4 (SAS Institute Inc), which is an automated randomization program. The researcher (ME) informed patients about their assignment to control or intervention group. All patients in both groups continued with their care as usual: regular visits, treatment at the outpatient clinics, and standardized cardiovascular risk management, which contained an evaluation of cardiovascular risk factors, including feedback to optimize lifestyle. In addition to the care as usual, patients in the intervention group received 12 months access to the intervention (October 2015 until October 2016) directly after randomization.

### Intervention

The Vascular View program was systematically developed in collaboration with CVD patients and health care professionals [20]. By defining performance and change objectives in conformity with the IM steps [19], 6 topics were distinguished and incorporated into the modules included in Vascular View: (1) coping with CVD, (2) setting boundaries in daily life, (3) lifestyle in general with specific attention to tobacco and harmful alcohol use, (4) healthy nutrition, (5) being physically active in a healthy way, and (6) interaction with health care professionals. Moreover, patients had access to two diaries (exercise and nutrition) in which patients could register their behavior to get insight into their exercise and nutrition routines. Each module comprised 3 or 4 sessions, which were personalized and supported by written information, tailored feedback, quotes from and videos of patients with CVD, pictures, and exercises. The Vascular View program started with information about the content and objectives of the 6 modules. The program was unguided but patients could complete an assessment and receive tailored advice about which of the 6 available modules was recommended for them.

Patients could visit the web-based self-management program and different modules as often as they wanted. Three groups were determined to give insight in the use of the program: nonusers, minimal users, or frequent users. Nonusers were patients that never or only once visited the program, minimal users visited the program 2 to 20 times, and frequent users visited more than 20 times. A detailed description of Vascular View, implementation, and the process evaluation is described elsewhere [20], and an overview of the program can be seen in Table 1.

### Measurements and Outcomes

#### Baseline Characteristics

All patients who completed the baseline questionnaire between August 2015 and October 2015 received a questionnaire after 6 months (T1) and after 12 months (T2). At baseline, the following demographic and disease-related characteristics were collected: age, sex, educational level, work participation, cultural background, diagnosis and comorbidity, duration of illness, body weight, height, computer use, and experience with rehabilitation programs. Patient-reported outcome measurements were assessed at baseline and during follow-ups (T1 and T2). These outcomes could be distinguished in generic and module-specific outcomes (Table 1). The great amount of outcomes resulted in a long questionnaire with a great demand of time on participants. Therefore, the questionnaire at T1 was shortened by omitting two questionnaires: the Illness Perception Questionnaire (IPQ) and Rand-36. When patients preferred a paper questionnaire, a version was sent by post.

#### General Patient-Reported Outcome Measurements

Patient’s illness attributions were assessed by the “causes of my illness” section on the IPQ. It contains 18 items measured on a 5-point Likert scale measuring four dimensions: psychological attributions, risk factors, immunity, and accident or chance. A higher score indicates a higher level of attribution to the dimension [23].

The patient’s general health status was measured with the Rand-36, consisting of 36 items measuring 8 dimensions: physical functioning, social functioning, physical role limitations, emotional role limitations, mental health, vitality, pain, and general health perception [24]. The subscales physical and emotional role limitations have dichotomous items. The other subscales contained Likert scale items, with a higher score indicating better perceived health-related quality of life. All subscale scores were transformed to a 0-100 point scale.

The Patient Activation Measure (PAM-13), which includes statements about an individual’s knowledge, confidence, and skills for self-management of their chronic illness behavior and the level of activation, was used to measure participants’ self-management ability. The PAM-13 includes 13 items on a 5-point scale with a higher score indicating a higher level of patient activation [25,26].

No validated questionnaire was available that corresponded to the aims of the intervention; therefore, patient’s self-efficacy was measured with a self-developed questionnaire (see Multimedia Appendix 1 for an overview of the scales and examples of items). The aim of this questionnaire was to measure how confident patients felt about self-managing CVD based on the performance objectives and corresponding determinants (step 2 of the IM framework). Four patients from the expert group, who were involved in the development of the Vascular View-program [20], were asked to participate in the Think Aloud procedure, a technique used to evaluate the questionnaire [27]. The final questionnaire included 26 items measuring 8 subscales. The items were scored on a 4-point scale with a higher score indicating a higher level of confidence about self-managing CVD. For each subscale, Cronbach 𝛼 was calculated using the baseline, 6-month, and 12-month data of both groups (control and intervention). Each subscale is related to a module in the Vascular View program (see Table 1): acceptance (Cronbach 𝛼=.81), social environment (Cronbach 𝛼=.87), interaction with professionals (Cronbach 𝛼=.85), physical activity (Cronbach 𝛼=.83), diet (Cronbach 𝛼=.84), smoking (Cronbach 𝛼=.83), alcohol (Cronbach 𝛼=.90), and setting boundaries (Cronbach 𝛼=.78).

#### Module-Specific Patient-Reported Outcome Measurements

To measure patient attitudes toward their prescribed medicine, the first scale on the Beliefs about Medicines Questionnaire (BMQ) was used, which contains 10 items on a 5-point Likert scale. Two subscales (concerns and necessity) each contained 5 items that summed up to a scale score. A higher score reflects higher levels of concerns or feelings of necessity concerning the prescribed medicine [28,29].

Patient physical activity was measured with the International Physical Activity Questionnaire (IPAQ, short version) [30]. The IPAQ contains 7 questions divided in 3 subscales: walking, moderate intensity activity, and vigorous intensity activity. These subscales are described in minutes per week.

The Dutch Healthy Diet Index is a 34-item questionnaire to estimate adherence to the 2006 Dutch guidelines for a healthy diet, containing 8 components: vegetables, fruit, dietary fiber, fish, saturated fats, trans fats, natrium, and alcohol. Per component the score ranges between 0 and 10, resulting in a total score between 0 (no adherence) and 80 (complete adherence) [31].

Patient tobacco dependence was measured with the Fagerström Test for Nicotine Dependence. This questionnaire consists of 6 items resulting in a total score between 1 and 10, in which a higher score reflects more dependence of nicotine [32].

Alcohol use was measured by the 3-item Alcohol Use Disorders Identification Test with a total score from 0 to 12. A score of 5 or higher indicates the possibility of increasing risk and higher risk of alcohol drinking [33].

Patient interaction with health care professionals was measured with the Perceived Efficacy in Patient-Physician Interaction, which contains 5 items on a 5-point Likert scale that are summed to determine the total score. A higher score reflects more confidence of the patient in interactions with their physician [34,35].

#### Biomedical Measurements

Electronic patient dossiers were searched for biomedical data on two time points: April 2015 to December 2015 (baseline measurement) and April 2016 to March 2017 (12-month measurement). The search of biomedical data included weight, BMI, systolic and diastolic blood pressure, total cholesterol, low-density lipoprotein–cholesterol, high-density lipoprotein–cholesterol, triglycerides, and non–high-density lipoprotein–cholesterol.

### Statistical Analysis

For explorative RCTs such as this, sample sizes are not calculated based on formal power analyses. Therefore, a sample size of 200 patients was chosen for this trial, which was considered a sufficient size for a representation of the relevant variation in the target group. All quantitative data were analyzed using SPSS Statistics version 25 (IBM Corporation). Descriptive analyses were used to describe the control and intervention groups at baseline. The differences between patient characteristics in the intervention and control group were tested using *t* tests and chi-square tests. A *P* value of <.05 was determined as statistically significant in all analyses.

A linear mixed-model analysis with repeated measures on intention-to-treat (ITT) basis was used to determine the differences in outcome measures between the intervention and control group. In this model, the outcomes were the dependent variables and the patient was the random factor. The fixed factors were group (intervention/control) and time and the interaction between time and group. This method automatically uses the missing at random assumption to handle missing data.

Subsequently, a per-protocol analysis (PPA) was performed to compare the control and intervention group with only those patients who completed the treatment originally allocated. Patients in the control group were included if they completed the questionnaire at baseline and 6 and 12 months. Patients in the intervention group were included if they completed the questionnaire at baseline and 6 and 12 months and used Vascular View at least once. Moreover, the same tests and linear mixed-model analyses were used as described in the ITT section.

### Identifying Outcome Measures

To identify outcome measures most likely to capture the potential benefits, floor and ceiling effects were explored for all outcome measurements using Likert scales at baseline. A floor effect indicates that most of the participants score near the minimum score and a ceiling effect indicates that most participants score near the maximum score on a questionnaire.

In this calculation, we considered floor and ceiling effects exceeding 20% to be significant [36]. Thereby, spaghetti plots were used to analyze the changes between T0 and T2 for all individuals.

## Results

### Participants

**Figure 1:**
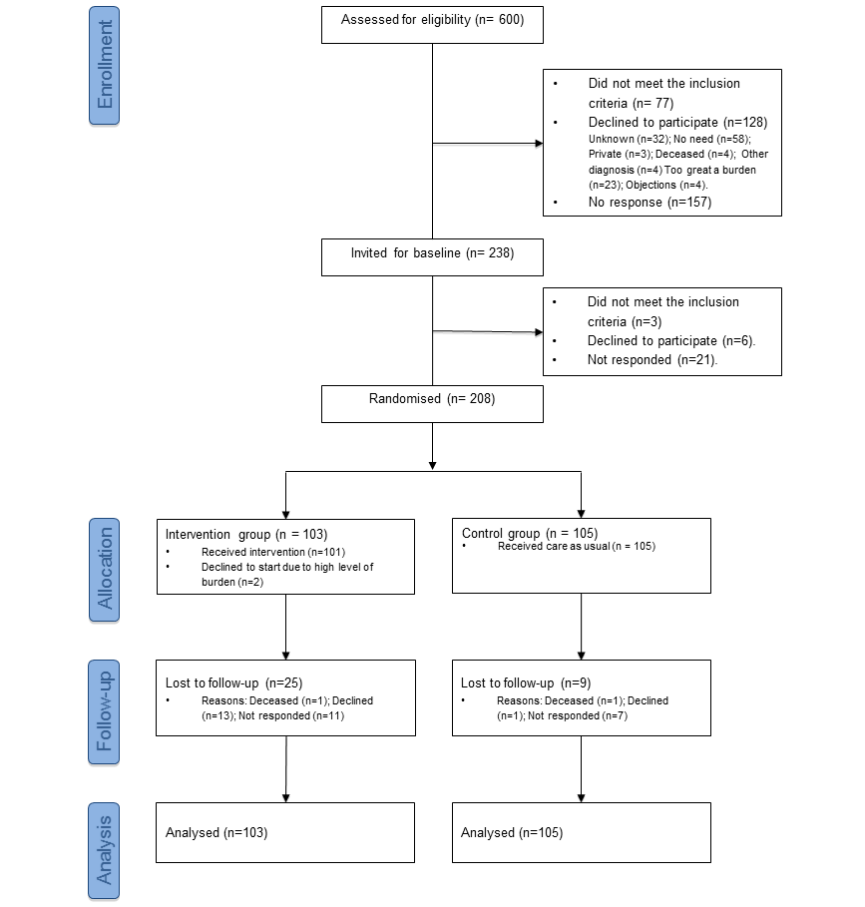
Flowchart of Vascular View study.

In total, 600 patients were eligible and invited by the medical specialist. Of these, 238 patients expressed interest, and 208 (87.4%) participated in the study (see Figure 1). Of the 30 patients who were not included, 3 did not meet the inclusion criteria, 6 declined, and 21 did not respond to the invitation. The 208 participating patients were randomized to the intervention (n=103) or control group (n=105) and stratified by CVD diagnosis. Two intervention group patients completed the baseline questionnaire but declined to participate in the intervention. At T1, 86 patients in the intervention group and 103 in the control group completed the questionnaire. At T2, 78 patients in the intervention group and 96 in the control group completed the questionnaire. More intervention group patients (25/103, 24.3%) compared with control group patients (9/105, 8.6%) were lost to follow-up.

All patients in the intervention group (n=101) had access to Vascular View, of which 37.6% (38/101) did not visit the program or only once, 27.7% (28/101) visited the program 2 to 20 times, and 34.7% (35/101) visited the program more than 20 times. The range of visits was between 1 and 43 visits per participant.

### Outcomes of the Intention-To-Treat Analysis

#### Baseline Characteristics of Patients

Demographic and disease-related characteristics at baseline were compared for the control and intervention group, as shown in Table 2. The mean age of the patients in the intervention group was 63.3 (SD 10.0) years and 63.7 (SD 9.8) years in the control group. Both groups had more men than women: 69 men in the control and 73 in the intervention group. There were no statistically significant differences between the control and intervention group.

**Table 2:** Intention-to-treat analysis: patient characteristics at baseline.

Characteristics		Control (n=105)		Intervention (n=103)	
Sex (male), n (%)		69 (66.0)		73 (71.0)	
Age in years, mean (SD)		63.7 (9.8)		63.3 (10.0)	
BMI (kg/m^2^), mean (SD)		28.4 (4.8)		27.2 (4.9)	
Disease duration, mean (SD)		4.9 (8.2)		4.3 (7.7)	
**Education level, n (%)**					
	Low		24 (22.9)		17 (16.5)
	Intermediate		39 (37.1)		34 (33.0)
	High		42 (40.0)		52 (50.5)
Work participation (yes), n (%)		35 (33.3)		40 (38.8)	
**Diagnosis group, n (%)**					
	Myocardial infarction		57 (54.3)		58 (56.3)
	Cerebrovascular disease (stroke included)		35 (33.3)		33 (32.0)
	Peripheral artery disease		13 (12.4)		12 (11.7)
Comorbidity within CVD^a^ (yes), n (%)		21 (20.0)		18 (17.5)	
Comorbidity, other (yes), n (%)		32 (30.5)		30 (32.0)	
Cultural background (Dutch), n (%)		101 (96.2)		100 (97.1)	

^a^CVD: cardiovascular disease.

#### Biomedical Measurements

An overview of the mean scores and standard deviations of patients in the intervention and control group at baseline and after 12 months is presented in Table 3. No statistically significant differences were found between the control and intervention group.

The biomedical data show a high level of missing values in both the control and intervention group. For example, triglycerides show a large group of missing values: 45% of values are gathered at T2 compared with T0.

**Table 3:** Biometrics of control and intervention group.

Biometrics	Control group (n=105)						Intervention group (n=103)					
	T0^a^		T2^b^			T0				T2		
	n	mean (SD)		n	mean (SD)			n	mean (SD)		n	mean (SD)
Weight (kg)	81	86 (16)		56	90 (20)			84	85 (15)		46	86 (15)
BMI (kg/m^2^)	81	28.3 (4.5)		56	29.3 (5.3)			83	27.3 (4.0)		46	27.5 (4.3)
Systolic BP^c^ (mm Hg)	96	135 (19)		84	135 (15)			94	135 (17)		68	134 (18)
Diastolic BP (mm Hg)	96	79 (10)		84	77 (11)			94	79 (9)		68	77 (10)
Total cholesterol	87	4.2 (1.1)		55	4.0 (0.9)			83	4.3 (1.2)		47	4.2 (1.1)
LDL-C^d^	67	2.2 (0.8)		33	2.1 (0.8)			65	2.3 (0.8)		29	2.2 (0.9)
HDL-C^e^	87	1.2 (0.3)		54	1.2 (0.4)			83	1.1 (0.3)		47	1.2 (0.3)
Triglycerides	71	1.9 (1.6)		33	1.8 (1.4)			69	1.9 (1.2)		30	1.9 (1.3)
non–HDL-C	87	3.0 (1.1)		53	2.7 (0.9)			82	3.2 (1.1)		46	3.0 (1.1)

^a^T0: baseline.

^b^T2: 12 months.

^c^BP: blood pressure.

^d^LDL-C: low-density lipoprotein–cholesterol.

^e^HDL-C: high-density lipoprotein–cholesterol.

#### Patient Outcome Measurements at Baseline and Follow-Up

Multimedia Appendix 2 provides an overview of the mean scores and standard deviations of outcome measurements of the patients in the intervention and control group at baseline and after 6 and 12 months. At baseline, the intervention group had more patients who smoked (n=13) compared with the control group (n=5), which was a statistically significant difference (mean difference 0.4; *P*=.04). Thereby, patients in the intervention group scored at baseline significantly higher on self-management behavior (PAM-13) compared with patients in the control group (mean difference 4.4; *P*=.03).

The estimated differences and *P* values between the intervention and control group after ITT analysis at 6 and 12 months after baseline are presented in Multimedia Appendix 2. Six out of 29 variables in the intervention group and 13 out of 29 in the control group decreased over time. Two questionnaires showed significant effects in favor of the intervention group. First, the IPQ scores showed that intervention group patients attributed the cause of their disease more to risk factors and immunity factors after 12 months. Patients in the control group showed a decrease on attribution to risk factors and a small increase on immunity factors. Second, the intervention group showed a statistically significant decrease for the dependency of nicotine after 6 months (–1.55; *P*=.01) and after 12 months (–1.67; *P*=.01) with respect to T0.

### Outcomes Per-Protocol Analysis

#### Baseline Characteristics of Patients

There were no significant between-group differences found in the demographic and disease-related characteristics at baseline for the control and intervention group of the patients following the intervention per protocol (Table 4).

**Table 4:** Per-protocol analysis: patient characteristics at baseline.

Characteristics		Control (n=95)		Intervention (n=52)
Sex (male), n (%)		62 (65.3)		36 (69.2)
Age in years, mean (SD)		63.9 (9.4)		61.8 (9.3)
BMI (kg/m^2^), mean (SD)		28.5 (4.9)		27.2 (5.2)
Disease duration, mean (SD)		4.7 (7.9)		4.0 (8.2)
**Education level, n (%)**				
	Low	23 (24.2)	12 (23.1)	
	Intermediate	34 (35.8)	12 (23.1)	
	High	38 (40.0)	28 (53.8)	
Work participation (yes), n (%)		19 (36.5)		29 (30.5)
**Diagnosis group, n (%)**				
	Myocardial infarction	51 (53.7)	34 (65.4)	
	Cerebrovascular disease (stroke included)	32 (33.7)	14 (26.9)	
	Peripheral artery disease	12 (12.7)	1 (7.7)	
Comorbidity within CVD^a^ (yes), n (%)		19 (20.0)		6 (11.5)
Comorbidity, other (yes), n (%)		29 (30.5)		17 (32.7)
Cultural background (Dutch), n (%)		92 (96.8)		51 (98.1)

^a^CVD: cardiovascular disease.

#### Patient Outcome Measurements at Baseline and Follow-Up

Multimedia Appendix 3 gives an overview of the PPA with mean scores, standard deviations, effect sizes, and *P* values of outcome measurements of the patients in the intervention and control group at baseline and after 6 and 12 months. At baseline, patients in the intervention group scored significantly higher on self-management behavior (PAM-13) compared with patients in the control group (mean difference 5.6; *P*=.03). The subscale social environment showed a significantly higher score on self-efficacy for intervention group patients compared with the control group (mean difference 0.2; *P*=.04) at baseline.

The PPA showed effects on the same outcomes as the ITT analysis. The IPQ scores on risk factors (0.31; *P*=.02) and immunity factors (0.48; *P*<.001) showed a statistically significant difference between intervention and control group: the IPQ scores show that after 12 months, patients in the intervention group attributed the cause of their disease more to risk factors and immunity factors. Patients in the control group showed a decrease on attribution to risk factors and no change on immunity factors. Patients in the intervention group decreased the dependency of nicotine significantly toward the control group after 6 months (–1.87; *P*=.01) and after 12 months (–1.72; *P*=.02).

### Suitable Outcome Measures

The percentages of patients scoring 0 (floor effects) or full marks (ceiling effects) on the 10 outcome measures were assessed at baseline. Significant floor effects were seen on the subscales IPQ psychological attributions (22.0%), IPQ immunity (35.3%), and IPQ accident or chance (26.6%). Significant ceiling effects were noticed on the subscales Rand social functioning (29.3%), Rand bodily pain (29.3%), patient self-efficacy with a self-constructed 26-item questionnaire (SE) interaction (26.9%), SE physical activity (33.8%), SE diet (30.9%), SE alcohol (35.2%), and SE setting boundaries (23.7%).

## Discussion

### Principal Findings

This explorative RCT aimed to evaluate the potential effectiveness and effect sizes of the Vascular View program on 10 patient outcome measures and identify the outcome measures most likely to capture potential benefits. The evaluation of potential effectiveness of Vascular View showed significant effects for illness attribution and nicotine dependence. At 12 months, patients in the intervention group attributed the cause of their diseases more often to risk factors and immunity factors than patients in the control group. Intervention group patients showed less dependency of nicotine after 6 and 12 months compared with the control group. It should be noted that we are not convinced that this effect was clinically meaningful due to the small number of participants who stopped or decreased smoking and because the effect is partly due to a high dropout of smokers in the intervention group (4 out of 13) compared with no dropout in the control group and one patient in the control group who started smoking after the baseline measure. Although the other outcomes showed no statistically significant differences between the intervention and control group, there seems to be a positive trend in the improvement of outcome measurements in favor of the intervention group. Overall, we did not expect to find a nonconvincing trend regarding the efficacy of Vascular View, since the theory-based intervention was thoroughly designed with patients and health care professionals on the basis of patient support needs according to the IM steps [19]. Furthermore, the outcome measures were selected carefully and in line with the objectives of the intervention. Randomization was successful, and the number of missing values was limited.

To our knowledge, Vascular View is the first web-based self-management program for secondary care patients with CVD that aims to improve so many components and in which patients can decide which modules they want to use. Other (effective) eHealth interventions for CVD prevention focused primarily on one or two risk factors (eg, physical activity [15] and smoking cessation [37]). Results of web-based self-management programs with multiple components in other diseases show comparable results [38-40]. Vascular View tried to guide patients by using a questionnaire to advise them in choosing courses in the welcome module and lifestyle module [20]. However, the large amount of topics might have resulted in an overload of information and subsequently demotivation and minimal use of the program. The lack of efficacy of the Vascular View program is not in line with studies that show the potential of eHealth interventions for secondary prevention of CVD [12-16]. For example, Vascular View addressed patients’ intrinsic processes through the determinants, which is seen as a successful strategy for face-to-face self-management programs [41]. However, it is hard to compare studies on eHealth self-management programs because of the various eHealth approaches and outcome measures available. Review studies show a large diversity in studies, looking at outcome measures, use of eHealth interventions, implementation, etc.

A discrepancy between needed self-management support by patients and provided self-management support by nurses is familiar in health care. Vascular View is an unguided self-management program in which nurses did not discuss the Vascular View program with patients, the program was not part of consultation, and nurses did not encourage patients to continue use of the program. The question should be raised whether use of the intervention should be supported and perhaps even used as a partial replacement of usual care. Providing self-management support is a core task of nurses in outpatient clinics, and patients expect health care professionals to fulfill a comprehensive role [42]. However, nurses seem to experience barriers in discussing all self-management categories (symptom management, treatment, biomedical cardiovascular risk factors, psychosocial consequences, and lifestyle changes). Physical components are often discussed, but psychological components are left behind [43]. Web-based self-management interventions could be a way to improve self-management support by focusing on the patient instead of the health care professional. More research needs to be conducted to determine the balance between support by the nurse and a self-guided self-management program.

Only half of the intervention group (n=51) adhered to the intervention: they completed all questionnaires and used Vascular View once or more often. Moreover, 38% did not use the program or used it only once. Although a previous questionnaire study showed that 77% of patients with CVD were interested in support through the internet [11], it seems to be important to match expectations of patients with the online program [44]. When patient expectations did not line up with the online program, patients refused to complete the measurements and revisit the online program [44]. Three striking differences between patients in the ITT and PPA give insight in the difference between users and nonusers. First, mostly patients with an intermediate education level drop out (difference between ITT and PPA: 9.9%) and a relatively high number of patients with a lower level of education used the program (difference between ITT and PPA: 6.6%). These results are contrary to other research suggesting that lower educational levels are a barrier for eHealth use [45]. Second, the percentage of patients with myocardial infarction is higher in the intervention group of the PPA (65.4%) compared with the ITT (56.3%). This suggests a higher need for self-management support in patients with a myocardial infarction diagnosis. Third, the Vascular View program seems to be more popular in patients scoring higher on patient-related outcomes such as self-management, physical activity, quality of life, diet, nicotine adherence, and alcohol adherence.Improvement is more difficult to achieve in this group.

The second aim was to identify the outcome measures most likely to capture the potential benefits. Most of the chosen outcome measures were likely to capture the potential effect, although floor and ceiling effects were seen in the IPQ, Rand-36, and patient’s self-efficacy questionnaire. Patient’s illness attributions (IPQ) [23] showed a right-skewed distribution, meaning that more than 20% of the patients attributed their disease not to psychological factors, immunity, accident, or chance at all. However, effects after 12 months were seen on the IPQ since the intervention group increased attribution to psychological factors, immunity, and risk factors. In spite of the floor effects, the IPQ is a valuable measurement to give insight in the efficacy of the Vascular View program. A left-skewed distribution was found in 2 subscales of the Rand-36 [24] (social functioning and bodily pain), indicating that more than 20% of the patients already experienced a high quality of life. Since CVD patients show relatively low pain levels and physical symptoms, for future research we suggest an instrument with a higher sensitivity such as the Seattle Angina Questionnaire [46,47]. The self-efficacy questionnaire also showed a left-skewed distribution, suggesting high self-efficacy on interaction, physical activity, diet, alcohol, and setting boundaries. More research should be conducted to determine the reliability and validity of this self-developed questionnaire. To conclude, a more sensitive instrument for quality of life is suggested for future research. All other questionnaires were sensitive to measure change in this population.

### Limitations

We believe this explorative RCT has numerous methodological strengths, although some limitations need to be mentioned. First, more patients in the intervention compared with the control group were lost to follow-up. However, we have not established that patients with specific characteristics dropped out. Second, this research was conducted in the outpatient clinic of a university hospital in which high quality of CVD care is already delivered, and therefore it may be harder to achieve improvement. Last, all patients in the outpatient group were informed but only the ones who were interested in the study were invited to participate. A limitation is that this might be a biased group because they were probably already interested in self-management.

### Recommendations for Clinical Practice and Research

For future studies, we recommend further studying patient self-management needs and the possibilities of tailoring eHealth. Although Vascular View was developed using IM on the basis of the support needs of patients with CVD [20], individual participating patients were not asked for their intentions to improve self-management and change their behavior and what kind of support needs they preferred. In our study, patients with higher starting levels (eg, self-management) were more likely to use the program. So, the readiness to change or phase of the disease might be predictors for using the program (and the needed support type). To conclude, to increase use and efficacy of eHealth programs, more insight into characteristics of patients who could benefit from web-based self-management programs is needed. A process evaluation will be conducted and published using the components of Saunders (fidelity, dose, reach, recruitment, and context) to get more insight into the low compliance with and noneffectiveness of the intervention and fine-tune the inclusion criteria. Furthermore, we believe that the program should be embedded in treatment and supplement self-management support provided by nurses. More research needs to be conducted to determine the balance between nurse support and self-guided self-management programs.

### Conclusions

This study contributes to our understanding of self-management support for patients with CVD using eHealth apps. Although we believe in the potential of the Vascular View program, there is no conclusive evidence for the efficacy. Using an unguided self-management program might not work for everyone, and the program might need to be embedded in health care more firmly. A detailed process evaluation of the program should be conducted to gain thorough insight into the working elements of the program, patient needs in eHealth, and the use of the program by patients. Finally, it should be investigated how Vascular View can be more tailored to the patient needs and become more embedded in treatment.

## Appendix

Multimedia Appendix 1 Self-efficacy questionnaire.

Multimedia Appendix 2 Intention-to-treat analysis: mean scores, standard deviation, effect sizes and *P* values of the outcome measures.

Multimedia Appendix 3 Per-protocol analysis: mean scores, standard deviation, effect sizes and *P* values of the outcome measures.

Multimedia Appendix 4 CONSORT-eHEALTH checklist (v.1.6.1).

## Abbreviations

BCT: behavior change technique
BMQ: Beliefs about Medicines Questionnaire
CVD: cardiovascular disease
eHealth: electronic health
IM: intervention mapping
IPAQ: International Physical Activity Questionnaire
IPQ: Illness Perception Questionnaire
ITT: intention to treat
mHealth: mobile health
PAM-13: Patient Activation Measure
PPA: per-protocol analysis
RCT: randomized controlled trial
SE: patient self-efficacy with a self-constructed 26-item questionnaire
