# Supplementary material for: Evaluation of a Web-Based Self-Management Program for Patients With Cardiovascular Disease: Explorative Randomized Controlled Trial
Source: J Med Internet Res. 2020 Jul 24;22(7):e17422. doi: 10.2196/17422 (PMC7414414; doi:10.2196/17422)
Supplement: Multimedia Appendix 1 [file jmir_v22i7e17422_app1.docx]

**Appendix 1: Self-efficacy questionnaire**

Underneath, an overview of the scales, number of items per scale, and examples per scale of the self-efficacy questionnaire are provided.

| **Scale** | **Items (n)** | **Examples** |
| --- | --- | --- |
| SE Acceptation | 7 | Item 2: I am able to adapt my life to my changed possibilities as a result of my condition.  Item 4: I am able to deal with pain associated with the condition. Item 12: I am able to find other forms of intimacy/sexuality after the diagnosis of my cardiovascular disease. |
| SE Social environment | 6 | Item 6: I am able to express emotional changes, as a result of my condition, to others (e.g. experienced fear, sadness, insecurity, guilt). Item 8: I am able to discuss overprotection with my loved ones. |
| SE Interaction | 3 | Item 25: I am able to indicate to the healthcare professional when the course of a conversation feels not pleasant/respectful. |
| SE Physical activity | 2 | Item 19: I am able to maintain the changes I have made to improve my physical activity. |
| SE Diet | 2 | Item 15: I am able to eat and drink healthier. |
| SE Smoking | 2 | Item 22: I am able to keep smoking cessation in the future. |
| SE Alcohol | 2 | Item 16: I am able to drink less alcohol or stop using alcohol. |
| SE Setting boundaries | 2 | Item 18: I am able to set my limits (for example: say “no”). |
