# Supplementary material for: Evaluation of a Web-Based Self-Management Program for Patients With Cardiovascular Disease: Explorative Randomized Controlled Trial
Source: J Med Internet Res. 2020 Jul 24;22(7):e17422. doi: 10.2196/17422 (PMC7414414; doi:10.2196/17422)
Supplement: Multimedia Appendix 2 [file jmir_v22i7e17422_app2.docx]

Multimedia Appendix 2. Intention-to-treat analysis: mean scores, standard deviation, effect sizes and *P* values of the outcome measures.

| Patient outcomes | Range |  | T0 | | T1 | | T2 | | 6-months | 12-months |
| --- | --- | --- | --- | --- | --- | --- | --- | --- | --- | --- |
|  | Min-max |  | n | Mean (SD) | n | Mean (SD) | n | Mean (SD) | Effect (p) | Effect (p) |
| IPQ Psychological attributions | 1-5 | C | 105 | 2.1 (1.0) |  |  | 95 | 1.9 (0.9) |  | 0.26 (.05) |
|  |  | I | 103 | 2.0 (0.8) |  |  | 78 | 2.0 (0.8) |  |  |
| IPQ Risk factors | 1-5 | C | 105 | 2.2 (0.7) |  |  | 94 | 2.1 (0.7) |  | 0.33 (.00)* |
|  |  | I | 103 | 2.0 (0.6) |  |  | 78 | 2.2 (0.7) |  |  |
| IPQ Immunity | 1-5 | C | 104 | 1.8 (0.8) |  |  | 94 | 1.9 (0.9) |  | 0.37 (.01)* |
|  |  | I | 103 | 1.7 (0.7) |  |  | 78 | 2.1 (0.8) |  |  |
| IPQ Accident or chance | 1-5 | C | 104 | 2.2 (1.0) |  |  | 94 | 2.1 (0.9) |  | 0.21 (.16) |
|  |  | I | 103 | 2.1 (0.8) |  |  | 77 | 2.2 (0.7) |  |  |
| RAND Physical functioning | 0-100 | C | 105 | 67.3 (26.7) |  |  | 96 | 67.0 (26.6) |  | -2.28 (.33) |
|  |  | I | 103 | 70.2 (26.2) |  |  | 78 | 69.1 (28.1) |  |  |
| RAND Social functioning | 0-100 | C | 105 | 74.2 (24.0) |  |  | 96 | 77.6 (20.4) |  | -3.59 (.24) |
|  |  | I | 103 | 75.4 (24.5) |  |  | 78 | 75.6 (26.2) |  |  |
| RAND Role physical | 0-100 | C | 105 | 54.0 (44.1) |  |  | 95 | 54.5 (44.2) |  | 0.09 (.99) |
|  |  | I | 103 | 58.7 (41.2) |  |  | 78 | 61.2 (42.8) |  |  |
| RAND Role emotional | 0-100 | C | 105 | 78.1 (37.8) |  |  | 95 | 73.7 (39.2) |  | 4.66 (.38) |
|  |  | I | 103 | 77.7 (36.6) |  |  | 78 | 81.6 (33.0) |  |  |
| RAND Mental health | 0-100 | C | 105 | 76.3 (15.6) |  |  | 96 | 75.4 (14.7) |  | 1.51 (.38) |
|  |  | I | 103 | 76.4 (16.2) |  |  | 78 | 77.6 (16.7) |  |  |
| RAND Vitality | 0-100 | C | 105 | 56.7 (20.2) |  |  | 96 | 58.5 (18.9) |  | -1.24 (.58) |
|  |  | I | 103 | 58.4 (20.3) |  |  | 78 | 59.4 (18.5) |  |  |
| RAND Bodily pain | 0-100 | C | 105 | 72.3 (23.4) |  |  | 96 | 72.4 (24.8) |  | -1.22 (.69) |
|  |  | I | 103 | 76.9 (23.5) |  |  | 78 | 76.6 (24.7) |  |  |
| RAND General health | 0-100 | C | 105 | 54.7 (19.3) |  |  | 96 | 52.8 (20.1) |  | 2.91 (.18) |
|  |  | I | 103 | 53.8 (19.3) |  |  | 78 | 56.0 (21.3) |  |  |
| RAND Health change | 0-100 | C | 105 | 46.0 (26.7) |  |  | 96 | 52.6 (21.3) |  | -1.70 (.71) |
|  |  | I | 103 | 51.2 (24.9) |  |  | 78 | 57.1 (26.7) |  |  |
| PAM-13 | 10-65 | C | 105 | 56.2 (13.9) | 103 | 57.7 (13.6) | 96 | 56.4 (15.7) | 0.32 (.88) | 1.03 (.63) |
|  |  | I | 103 | 60.6 (14.6) | 86 | 62.5 (15.7) | 78 | 61.9 (15.3) |  |  |
| SE Acceptation | 1-4 | C | 104 | 3.1 (0.5) | 103 | 3.1 (0.5) | 96 | 3.2 (0.5) | -0.06 (.33) | -0.02 (.78) |
|  |  | I | 103 | 3.2 (0.5) | 86 | 3.2 (0.5) | 78 | 3.3 (0.5) |  |  |
| SE Social environment | 1-4 | C | 104 | 3.0 (0.5) | 103 | 3.0 (0.6) | 96 | 3.1 (0.5) | 0.03 (.69) | 0.04 (.56) |
|  |  | I | 103 | 3.2 (0.6) | 86 | 3.2 (0.5) | 78 | 3.3 (0.6) |  |  |
| SE Interaction | 1-4 | C | 103 | 3.3 (0.6) | 103 | 3.2 (0.6) | 95 | 3.2 (0.5) | 0.06 (.42) | 0.16 (.06) |
|  |  | I | 103 | 3.3 (0.7) | 86 | 3.3 (0.6) | 78 | 3.4 (0.6) |  |  |
| SE Physical activity | 1-4 | C | 104 | 3.3 (0.6) | 103 | 3.2 (0.7) | 95 | 3.2 (0.7) | 0.08 (.32) | 0.04 (.60) |
|  |  | I | 103 | 3.4 (0.6) | 86 | 3.4 (0.6) | 78 | 3.3 (0.7) |  |  |
| SE Diet | 1-4 | C | 104 | 3.3 (0.6) | 103 | 3.2 (0.6) | 95 | 3.2 (0.7) | 0.07 (.43) | 0.03 (.75) |
|  |  | I | 103 | 3.4 (0.6) | 86 | 3.3 (0.6) | 78 | 3.3 (0.6) |  |  |
| SE Smoking | 1-4 | C | 5 | 2.1 (0.7) | 5 | 2.1 (0.9) | 5 | 2.5 (0.7) | -0.33 (.35) | -0.73 (.05) |
|  |  | I | 13 | 2.4 (0.5) | 11 | 2.1 (0.7) | 8 | 2.1 (0.5) |  |  |
| SE Alcohol | 1-4 | C | 61 | 3.3 (0.7) | 56 | 3.1 (0.6) | 52 | 3.1 (0.6) | 0.11 (.26) | 0.15 (.12) |
|  |  | I | 67 | 3.3 (0.7) | 51 | 3.3 (0.6) | 48 | 3.4 (0.6) |  |  |
| SE Setting boundaries | 1-4 | C | 104 | 3.1 (0.6) | 103 | 3.1 (0.7) | 95 | 3.1 (0.6) | 0.01 (.93) | 0.02 (.87) |
|  |  | I | 103 | 3.2 (0.8) | 86 | 3.1 (0.7) | 78 | 3.1 (0.8) |  |  |
| PEPPI-5 | 5-25 | C | 104 | 20.1 (3.3) | 103 | 19.3 (3.5) | 96 | 20.1 (3.3) | 0.61 (.16) | 0.33 (.47) |
|  |  | I | 103 | 20.4 (3.4) | 86 | 20.2 (3.1) | 78 | 20.8 (3.0) |  |  |
| BMQ Concerns | 5-25 | C | 104 | 14.2 (3.9) | 103 | 13.9 (3.9) | 96 | 13.6 (3.6) | 0.45 (.49) | 0.40 (.99) |
|  |  | I | 103 | 13.8 (3.5) | 86 | 13.9 (3.6) | 78 | 13.5 (3.6) |  |  |
| BMQ Necessity | 5-25 | C | 104 | 18.3 (3.0) | 103 | 17.8 (3.1) | 96 | 17.6 (3.3) | 0.29 (.31) | 0.00 (.38) |
|  |  | I | 103 | 18.0 (4.0) | 86 | 18.0 (3.6) | 78 | 17.8 (4.2) |  |  |
| IPAQ Walking | m/w | C | 100 | 339 (319) | 99 | 350 (348) | 92 | 354 (340) | -33.2 (.50) | -17.5 (.73) |
|  |  | I | 97 | 325 (322) | 81 | 323 (298) | 74 | 330 (262) |  |  |
| IPAQ Moderate | m/w | C | 96 | 373 (364) | 96 | 348 (333) | 92 | 336 (300) | -.08 (.99) | 26.0 (.64) |
|  |  | I | 98 | 333 (318) | 79 | 318 (312) | 76 | 333 (276) |  |  |
| IPAQ Vigorous | m/w | C | 99 | 114 (170) | 100 | 228 (299) | 96 | 161 (214) | -50.7 (.19) | -47.5 (.23) |
|  |  | I | 100 | 145 (197) | 82 | 218 (275) | 76 | 155 (210) |  |  |
| DHD-index | 0-80 | C | 105 | 57.1 (11.1) | 103 | 57.1 (11.3) | 96 | 57.0 (10.8) | 1.24 (.29) | -0.45 (.72) |
|  |  | I | 103 | 56.0 (12.0) | 86 | 56.5 (10.2) | 78 | 55.5 (9.3) |  |  |
| FTND | 1-10 | C | 5 | 3.4 (2.2) | 5 | 5.2 (2.7) | 5 | 5.0 (2.7) | -1.55 (.01) | -1.67 (.01)* |
|  |  | I | 13 | 3.8 (1.6) | 11 | 3.5 (1.9) | 8 | 3.3 (2.1) |  |  |
| AUDIT | 0-12 | C | 62 | 4.0 (1.8) | 56 | 3.9 (1.8) | 52 | 3.9 (1.5) | 0.09 (.67) | 0.00 (.99) |
|  |  | I | 67 | 4.2 (1.9) | 51 | 4.2 (1.7) | 48 | 4.1 (1.8) |  |  |

IPQ: Illness Perception Questionnaire; RAND-36: patient’s health-related quality of life; PAM-13: Patient Activation Measurement; SE: patient’s self-efficacy with a self-constructed 26-item questionnaire; PEPPI-5: Perceived Efficacy in Patient-Physician Interactions tool; BMQ: Beliefs Medicine Questionnaire; IPAQ: International Physical Activity Questionnaire; DHD-Index: patient’s healthy eating habits with the Dutch Healthy Diet Index; FTND: Fagerström Test for Nicotine Dependence; AUDIT: Alcohol Use Disorders Identification Test
C: Control group; I: Intervention group
SD: Standard Deviation; m/w: Minutes per week
*Statistical significance (*P*<.05)
