# Supplementary material for: Evaluation of a Web-Based Self-Management Program for Patients With Cardiovascular Disease: Explorative Randomized Controlled Trial
Source: J Med Internet Res. 2020 Jul 24;22(7):e17422. doi: 10.2196/17422 (PMC7414414; doi:10.2196/17422)
Supplement: Multimedia Appendix 3 [file jmir_v22i7e17422_app3.docx]

Multimedia Appendix 3. Per-protocol analysis: mean scores, standard deviation, effect sizes and P values of the outcome measures.

| Patient outcomes | Range |  | T0 | | T1 | | T2 | | 6-months | 12-months |
| --- | --- | --- | --- | --- | --- | --- | --- | --- | --- | --- |
|  | Min-max |  | n | Mean (SD) | n | Mean (SD) | n | Mean (SD) | Effect (p) | Effect (p) |
| IPQ Psychological attributions | 1-5 | C | 95 | 2.1 (0.9) |  |  | 94 | 2.0 (0.9) |  | 0.24 (.14) |
|  |  | I | 52 | 1.9 (0.9) |  |  | 52 | 2.0 (0.9) |  |  |
| IPQ Risk factors | 1-5 | C | 95 | 2.2 (0.7) |  |  | 93 | 2.1 (0.7) |  | 0.31 (.02)* |
|  |  | I | 52 | 2.0 (0.6) |  |  | 52 | 2.2 (0.7) |  |  |
| IPQ Immunity | 1-5 | C | 94 | 1.8 (0.8) |  |  | 93 | 1.8 (0.9) |  | 0.48 (.00)* |
|  |  | I | 52 | 1.6 (0.7) |  |  | 52 | 2.1 (0.9) |  |  |
| IPQ Accident or chance | 1-5 | C | 94 | 2.1 (0.9) |  |  | 93 | 2.1 (1.0) |  | 0.20 (.24) |
|  |  | I | 52 | 2.0 (0.9) |  |  | 52 | 2.3 (0.7) |  |  |
| RAND Physical functioning | 0-100 | C | 95 | 66.3 (27.3) |  |  | 95 | 67.3 (26.6) |  | -1.00 (.71) |
|  |  | I | 52 | 72.2 (26.2) |  |  | 52 | 72.2 (26.3) |  |  |
| RAND Social functioning | 0-100 | C | 95 | 74.1 (24.3) |  |  | 95 | 77.8 (20.4) |  | -0.56 (.87) |
|  |  | I | 52 | 74.8 (27.3) |  |  | 52 | 77.9 (25.3) |  |  |
| RAND Role physical | 0-100 | C | 95 | 52.9 (44.0) |  |  | 94 | 54.8 (44.3) |  | 1.22 (.87) |
|  |  | I | 52 | 59.1 (41.7) |  |  | 52 | 62.5 (42.4) |  |  |
| RAND Role emotional | 0-100 | C | 95 | 78.2 (37.6) |  |  | 94 | 73.8 (39.4) |  | 3.90 (.54) |
|  |  | I | 52 | 85.3 (29.1) |  |  | 52 | 84.6 (29.9) |  |  |
| RAND Mental health | 0-100 | C | 95 | 76.5 (15.8) |  |  | 95 | 75.4 (14.7) |  | 1.71 (.39) |
|  |  | I | 52 | 77.1 (15.0) |  |  | 52 | 77.7 (16.6) |  |  |
| RAND Vitality | 0-100 | C | 95 | 57.1 (18.9) |  |  | 95 | 58.7 (18.9) |  | 0.46 (.86) |
|  |  | I | 52 | 58.0 (19.8) |  |  | 52 | 60.0 (19.1) |  |  |
| RAND Bodily pain | 0-100 | C | 95 | 71.7 (23.5) |  |  | 95 | 72.2 (24.8) |  | 0.09 (.98) |
|  |  | I | 52 | 76.6 (23.0) |  |  | 52 | 77.2 (24.6) |  |  |
| RAND General health | 0-100 | C | 95 | 54.6 (19.2) |  |  | 95 | 53.1 (20.0) |  | 1.91 (.46) |
|  |  | I | 52 | 55.2 (20.1) |  |  | 52 | 55.6 (22.4) |  |  |
| RAND Health change | 0-100 | C | 95 | 46.3 (26.5) |  |  | 95 | 52.9 (27.7) |  | 0.63 (.90) |
|  |  | I | 52 | 54.8 (24.8) |  |  | 52 | 62.0 (26.0) |  |  |
| PAM-13 | 10-65 | C | 95 | 56.3 (14.0) | 95 | 58.3 (56.4) | 95 | 56.4 (15.8) | 1.70 (.51) | 1.48 (.57) |
|  |  | I | 52 | 61.9 (16.8) | 52 | 65.6 (60.0) | 52 | 63.4 (16.9) |  |  |
| SE Acceptation | 1-4 | C | 94 | 3.1 (0.5) | 95 | 3.1 (0.5) | 95 | 3.2 (0.5) | -0.03 (.74) | 0.03 (.68) |
|  |  | I | 52 | 3.2 (0.5) | 52 | 3.2 (0.5) | 52 | 3.3 (0.6) |  |  |
| SE Social environment | 1-4 | C | 94 | 3.1 (0.5) | 95 | 3.0 (0.5) | 95 | 3.1 (0.5) | -0.00 (.99) | -0.06 (.50) |
|  |  | I | 52 | 3.3 (0.6) | 52 | 3.2 (0.6) | 52 | 3.2 (0.7) |  |  |
| SE Interaction | 1-4 | C | 93 | 3.3 (0.6) | 95 | 3.2 90.6) | 94 | 3.2 (0.5) | 0.09 (.37) | 0.16 (.10) |
|  |  | I | 52 | 3.3 (0.6) | 52 | 3.4 (0.6) | 52 | 3.4 (0.6) |  |  |
| SE Physical activity | 1-4 | C | 94 | 3.3 (0.6) | 95 | 3.2 (0.7) | 94 | 3.2 (0.7) | 0.04 (.71) | -0.03 (.77) |
|  |  | I | 52 | 3.5 (0.6) | 52 | 3.5 (0.6) | 52 | 3.4 (0.7) |  |  |
| SE Diet | 1-4 | C | 94 | 3.3 (0.6) | 95 | 3.2 (0.6) | 94 | 3.2 (0.7) | 0.09 (.37) | -0.03 (.73) |
|  |  | I | 52 | 3.5 (0.6) | 52 | 3.4 (0.6) | 52 | 3.3 (0.7) |  |  |
| SE Smoking | 1-4 | C | 6 | 2.3 (0.6) | 5 | 2.1 (0.9) | 5 | 2.5 (0.7) | -0.31 (.51) | -0.66 (.19) |
|  |  | I | 5 | 2.0 (0.7) | 6 | 2.0 (0.9) | 5 | 2.1 (0.7) |  |  |
| SE Alcohol | 1-4 | C | 56 | 3.3 (0.7) | 52 | 3.1 (0.6) | 52 | 3.1 (0.6) | 0.18 (.11) | 0.20 (.07) |
|  |  | I | 34 | 3.3 (0.7) | 28 | 3.4 (0.6) | 31 | 3.5 (0.6) |  |  |
| SE Setting boundaries | 1-4 | C | 94 | 3.1 (0.6) | 95 | 3.1 (0.6) | 94 | 3.1 (0.6) | 0.06 (.59) | 0.06 (.56) |
|  |  | I | 52 | 3.1 (0.8) | 52 | 3.1 (0.7) | 52 | 3.1 (0.8) |  |  |
| PEPPI-5 | 5-25 | C | 94 | 20.2 93.3) | 95 | 19.4 (3.5) | 95 | 20.1 (3.3) | 0.96 (.06) | 0.95 (.06) |
|  |  | I | 52 | 20.2 (3.7) | 52 | 20.4 (3.2) | 52 | 21.1 (3.0) |  |  |
| BMQ Concerns | 5-25 | C | 94 | 14.0 (4.0) | 95 | 13.8 (3.9) | 95 | 13.6 (3.6) | 0.10 (.85) | 0.37 (0.49) |
|  |  | I | 52 | 13.9 (3.7) | 52 | 13.7 (3.7) | 52 | 13.1 (3.5) |  |  |
| BMQ Necessity | 5-25 | C | 94 | 18.3 (3.1) | 95 | 17.9 (3.1) | 95 | 17.6 (3.3) | 0.09 (.87) | -0.28 (.58) |
|  |  | I | 52 | 17.9 (4.1) | 52 | 17.6 (3.7) | 52 | 17.6 (4.4) |  |  |
| IPAQ Walking | m/w | C | 91 | 339 (320) | 91 | 350 (341) | 91 | 357 (341) | -37.9 (.53) | -10.8 (.86) |
|  |  | I | 48 | 341 (327) | 48 | 337 (330) | 48 | 362 (294) |  |  |
| IPAQ Moderate | m/w | C | 88 | 362 (354) | 89 | 348 (333) | 91 | 340 (300) | -31.5 (.63) | 17.38 (.79) |
|  |  | I | 50 | 385 (323) | 47 | 350 (302) | 50 | 385 (276) |  |  |
| IPAQ Vigorous | m/w | C | 91 | 117 (174) | 92 | 208 (271) | 95 | 161 (215) | -67.9 (.13) | -54.0 (.23) |
|  |  | I | 50 | 185 (211) | 50 | 211 (241) | 50 | 182 (217) |  |  |
| DHD-index | 0-80 | C | 95 | 57.2 (11.0) | 95 | 57.2 (11.4) | 95 | 57.0 (10.9) | 2.10 (.13) | -1.44 (.30) |
|  |  | I | 52 | 56.8 (11.5) | 52 | 58.9 (9.7) | 52 | 55.2 (9.9) |  |  |
| FTND | 1-10 | C | 5 | 3.4 (2.2) | 5 | 5.2 (2.7) | 5 | 5.0 (2.7) | -1.87 (.01) | -1.72 (.02)* |
|  |  | I | 6 | 4.0 (2.1) | 6 | 3.0 (2.3) | 5 | 2.8 (2.2) |  |  |
| AUDIT | 0-12 | C | 57 | 4.0 (1.7) | 52 | 4.0 (1.8) | 52 | 3.9 (1.5) | 0.08 (.78) | 0.00 (.97) |
|  |  | I | 34 | 3.9 (2.0) | 28 | 3.9 (1.7) | 31 | 4.0 (1.6) |  |  |

IPQ: Illness Perception Questionnaire; RAND-36: patient’s health-related quality of life; PAM-13: Patient Activation Measurement; SE: patient’s self-efficacy with a self-constructed 26-item questionnaire; PEPPI-5: Perceived Efficacy in Patient-Physician Interactions tool; BMQ: Beliefs Medicine Questionnaire; IPAQ: International Physical Activity Questionnaire; DHD-Index: patient’s healthy eating habits with the Dutch Healthy Diet Index; FTND: Fagerström Test for Nicotine Dependence; AUDIT: Alcohol Use Disorders Identification Test
C: Control group; I: Intervention group
SD: Standard Deviation; m/w: Minutes per week
*Statistical significance (*P*<.05)
